# Therapeutic Endoscopic Retrograde Cholangiopancreatography for Pediatric Hepato-Pancreato-Biliary Diseases: A Systematic Review and Meta-Analysis

**DOI:** 10.3389/fped.2022.915085

**Published:** 2022-06-30

**Authors:** Rongjuan Sun, Xiaodan Xu, Qipeng Zheng, Jianghua Zhan

**Affiliations:** ^1^Graduate School, Tianjin Medical University, Tianjin, China; ^2^Department of General Surgery, Tianjin Children’s Hospital, Tianjin, China

**Keywords:** cholangiopancreatography, endoscopic retrograde, therapeutics, pediatrics, hepatobiliary, pancreas, meta-analysis

## Abstract

**Background:**

Hepato-pancreato-biliary (HPB) disease has different causes and types between children and adults, which has been increasingly diagnosed in the pediatric group. Endoscopic retrograde cholangiopancreatography (ERCP) has been gradually considered as a therapeutic method in adults, while in pediatric patients, there are not many reports of its usage. This systematic review and meta-analysis aims to assess the use condition of therapeutic ERCP in the management of pediatric HPB diseases.

**Methods:**

This systematic literature search was conducted in the PubMed, Embase, Web of Science, and Cochrane library databases to identify all relevant articles published from inception to February 2022 that evaluated therapeutic ERCP in pediatric patients with HPB diseases. The researchers included studies in which patients were less than 18 years old and underwent therapeutic ERCP procedures. A random-effects model was used to analyze the usage rate of therapeutic ERCP procedures, procedural success rates, adverse event rates, and the rate of different therapeutic procedures. Subgroup analysis, sensitivity analysis, and meta-regression were conducted to analyze the source of heterogeneity.

**Results:**

A total of 33 articles were included. After homogenization, the overall use of therapeutic interventions accounts for 77% [95% confidence interval (CI) 74–81%] of all ERCP procedures. After excluding outlier studies, the estimation success rate of the therapeutic procedure is 74% (95% CI 69–79%), and adverse event rate is 8% (95% CI 6–10%). In our study, stent placement is the most common procedure, which makes up 75% (95% CI 65–86%) of all therapeutic procedures. In addition, the usage proportion of sphincterotomy (ST), stone extraction/removal, bougienage/balloon dilation is, respectively, 46% (95% CI 39–53%), 34% (95% CI 31–38%), and 26% (95% CI 22–29%).

**Conclusion:**

The ERCP procedure is gradually considered a therapeutic technique in pediatric patients, the proportion of therapeutic ERCP is 77% of total usage, which is increasing every year. Meanwhile, its success rate is relatively high. It reflects that this operation modality is promising in the treatment of HPB disorders and is gradually expanded as more branch technologies are being used. A variety of operations can be achieved through ERCP procedures, and more functions should be developed in the future.

**Systematic Review Registration:**

[https://www.crd.york.ac.uk/prospero/], identifier [CRD42022302911].

## Introduction

Hepato-pancreato-biliary (HPB) disease refers to the condition that affects the liver, pancreas, and biliary system. For adults, most are caused by chronic damage to the organ tissues, while in children, most have various genetic or hereditary causes. Traumatic HPB disruption and severe biliary complications (BC) after liver transplantation have a comparatively lower incidence rate in pediatric patients. Endoscopic retrograde cholangiopancreatography (ERCP) has traditionally been performed to diagnose adult HPB diseases. It has unique effectiveness in delineating ductal anatomy, which is especially beneficial in those patients with pancreaticobiliary maljunction (PBM) (1). With significant advancements in endoscopic techniques, many non-invasive diagnostic methods have replaced ERCP in pediatric populations (2). ERCP today is mainly restricted to therapeutic performance, which includes any interventional procedure performed in addition to a cholangiopancreatogram. Because of the difference in disease types, the indications of therapeutic ERCP between children and adults are significantly different. Children with HPB disease often experience acute abdominal pain, obstructive jaundice, abnormal liver function, and other fatal conditions. Prophylactic surgeries may lead to high-risk post-operative complications, and therapeutic ERCP can improve digestive juice drainage and resolve complications relatively safely. This requires pediatric surgeons to make efforts on establishing specific clinical and technical indications, preparing for the use of minimal invasive equipment as well as an effective procedure for children. Minimal invasive techniques including endoscopic surgery are gradually replacing invasive interventions such as bile duct exploration surgery.

Compared with the adult population, the therapeutic option of ERCP is relatively rare in most children’s hospitals. The usage of therapeutic ERCP specifically in pediatric HPB diseases has not been systematically evaluated. We carry out this systematic review and meta-analysis to comprehensively assess the use condition of therapeutic ERCP in the management of pediatric HPB diseases up to now. Specifically, this will expand the awareness of pediatric surgeons in the use of various minimally invasive techniques through ERCP to treat HPB diseases instead of surgery. What is more, it will contribute to the improvement of these therapeutic methods and enhance the utilization rate in this field.

## Methods

This systematic review protocol was prospectively registered (PROSPERO ID: CRD42022302911). The review is reported according to the preferred reporting items for systematic reviews and meta-analyses (PRISMA) statement (3).

### Search Strategy

This systematic literature search was conducted in the PubMed, Embase, Web of Science, and Cochrane library database to identify all relevant articles published from inception to February 2021 that evaluated therapeutic ERCP in pediatric patients with HPB diseases. Trial registry websites such as ClinicalTrials.gov and WHO ICTRP were also searched. The following terms were searched: (“Pediatrics”[Mesh] OR “Child”[Mesh] OR “Adolescent”[Mesh] OR “Infant”[Mesh] AND “Cholangiopancreatography, Endoscopic Retrograde”[Mesh] AND “Therapeutics”[Mesh]). HPB diseases were included in the search results. To be as comprehensive as possible, the search was not restricted to any study types. We also hand-searched the reference lists of candidate articles to find the articles that might have been missed during the literature search. Our whole search strategy for all databases is described in the Supplementary Material.

### Selection Criteria

Study inclusion criteria included the following: (1) the patients were less than 18 years old; (2) the patients underwent therapeutic ERCP procedures; (3) the patients all had HPB diseases; (4) the procedural success rate and adverse event rate were assessed in the studies; (5) they have detailed description and statistics of various specific treatment methods; and (6) they were randomized controlled trials, case-control studies, or cohort studies.

We excluded the studies that were (1) case reports and case series, which particularity cannot represent the general outcome, and their sampling population is too small to perform meta-analysis; (2) editorial letters, expert opinions, review papers, and meta-analysis as to avoid erroneous weighting toward more frequently cited articles; (3) conference abstracts which could not obtain their full study reports as their scientific rigorousness had not been peer-reviewed; (4) endoscopic nasobiliary drainage (ENBD) or endoscopic ultrasound-guided biliary drainage (EUS-BD) were the only therapeutic methods (If ERCP had been included in these studies, the data about ERCP alone was extracted); (5) pertinent data (such as the percentage of therapeutic interventions in all ERCP procedures, procedural success rates, and adverse event rates) were not available; (7) the cases included were not HPB diseases; and (6) not English-language articles.

If similar articles containing the same population or duplicate cases from a single center were available, we included the article that (1) contains the most comprehensive detail of study characteristics; (2) whose data set has the largest sample; (3) is the most recently published one for our review.

All articles searched through the four databases were imported into Endnote for screening. Two independent reviewers (RS and XX) screened all articles relevant to therapeutic ERCP for pediatric HPB diseases for methodological validity, and a third reviewer (QZ) resolved the discordance during the study. Titles, abstracts, and keywords were evaluated firstly, and full texts of the articles were retrieved and evaluated for eligibility after the initial screening. Two reviewers (RS and QZ) assessed the quality measures of the included studies, and discrepancies were adjudicated *via* consensus or referral to a third reviewer (XX).

### Data Extraction

Data from the included studies were extracted into a standard form, detailing the first author(s), publication year, country, study design, study population, number of therapeutic ERCP procedures, procedural success rates, and adverse event rates. The number or rate of different therapeutic procedures was also extracted. We contacted the authors of articles for confirmation or correction when there was missing or unclear information in the paper. Data extraction was carried out using Microsoft Excel (Microsoft, Redmond, WA, United States).

### Outcome Measures

On account of variations in study design and reporting among the included publications, we precluded the use of a standard definition for procedural success and post-ERCP adverse events in some specific studies to make a more comprehensive assessment of the outcome. In our review, procedural success was defined as the successful completion of the determined treatment of the desired endoscope therapeutic intervention (cannulation *via* the papilla to perform certain treatments successfully), and clinical condition improvement [relief of symptoms or improvement of biochemical tests: such as the decrease of total bilirubin, alanine transaminase, and γ-glutamyltranspeptidase (*p* < 0.01) in liver function tests (LFT)]. Adverse events were defined as overall complications (such as pancreatitis, perforation, and bleeding). In these cases, patients’ treatment should be altered, which means more diagnostic investigation, prescription of antibiotics, prolonged hospitalization, readmission, or even subsequent surgery was needed (4). Death is one of the extremes included.

### Bias Assessment

The probability of publication bias was assessed through the visual inspection of funnel plots and Egger’s regression intercept.

### Statistical Analysis

This is a meta-analysis of proportions, which was performed using the Metan program with STATA Version 16 (StataCorp LP, College Station, TX, United States). We provide an overall estimate of the usage percentage of therapeutic interventions in all ERCP procedures, success rates of therapeutic procedures, adverse event rates, and the distribution of different therapeutic interventions. Due to the significant variability between studies, a random-effects meta-analysis was chosen over a fixed-effects meta-analysis using the restricted maximum likelihood method. A total of 95% confidence intervals (CIs) were calculated initially for the rate of each statistical result from the studies. The *I*^2^ statistic was used to evaluate the variance attributable to heterogeneity between individual studies. *I*^2^ < 25% was considered no heterotrophy, *I*^2^ between 25 and 50% was defined as low heterotrophy, *I*^2^ between 50 and 75% was defined as moderate heterotrophy, and *I*^2^ > 75% was defined as high heterotropy (5).

To find out the source of high heterogeneity, secondary analyses were performed to further investigate the influencing factors. A subgroup analysis was performed based on various countries, types of diseases, the year of publication, and design of the study. A sensitivity analysis of all studies was also performed to exclude papers deemed to be outliers. Outlier papers were identified based on the findings from the inclusive overall estimation.

### Quality Assessment

The quality of all 33 included non-randomized studies was assessed according to the Newcastle–Ottawa scale (NOS) (Table 1). The NOS assessment tool is based on a star grading system, which includes three categorical criteria. According to this standard scoring algorithm, prospective and cross-sectional studies can award a maximum of nine scores and case-control studies can award a maximum of 10 scores. Studies that received a score of above six were deemed as high quality. Two reviewers (RS and XX) independently assessed the study quality using this format, and disagreements were solved by another author (JZ).

**TABLE 1:** The Newcastle–Ottawa scale (NOS) of studies included.

Study	Selection				Comparability		Outcome assessment			Total score
	1	2	3	4	1	2	1	2	3	
Barakat et al. (23)	+	+	+	+			+	+	+	7
Mercier et al. (11)	+		+		+		+	+	+	6
Lin et al. (24)	+	+	+	+			+	+	+	7
Asenov et al. (25)	+	+	+		+		+	+		6
Harputluoglu et al. (26)	+	+	+	+	+		+	+	+	8
Wen et al. (27)	+	+	+	+			+	+	+	7
Zeng et al. (28)	+	+	+		+		+	+	+	7
Kohoutova et al. (29)	+	+	+				+	+	+	6
Czubkowski et al. (13)	+		+	+	+		+	+	+	7
Rosen et al. (30)	+	+	+	+	+		+	+	+	8
Giefer and Kozarek (31)	+	+	+		+	+	+	+	+	8
Dechêne et al. (32)	+	+	+		+		+		+	5
Kargl et al. (33)	+	+	+				+	+	+	6
Agarwal et al. (34)	+	+	+		+		+	+	+	7
Oracz et al. (35)	+	+	+				+	+	+	6
Steen et al. (36)	+	+	+		+		+		+	6
Tsuchiya et al. (16)	+	+	+				+			5
Limketkai et al. (37)	+	+	+		+		+	+		6
Enestvedt et al. (38)	+	+	+				+	+	+	6
Otto et al. (39)	+	+	+	+	+		+		+	7
Berquist (40)	+	+	+		+		+			5
Otto et al. (41)	+	+	+	+			+	+	+	7
Jang et al. (42)	+	+	+		+		+	+	+	7
Li et al. (18)	+	+	+	+	+		+	+	+	8
Vegting et al. (43)	+	+	+		+		+		+	6
Issa et al. (17)	+	+	+				+	+	+	6
Rocca et al. (1)	+	+	+		+		+	+	+	7
Cheng et al. (44)	+	+	+	+	+		+	+	+	8
Varadarajulu et al. (45)	+	+	+	+			+	+	+	7
Pfau et al. (46)	+	+	+				+	+	+	6
Poddar et al. (47)	+	+	+		+		+	+	+	7
Hsu et al. (48)	+	+	+	+			+	+	+	7
Guelrud et al. (49)	+	+	+				+	+	+	6

*Selection:*

*1: Representativeness of the exposed cohort.*

*2: Selection of the non-exposed cohort.*

*3: Ascertainment of exposure.*

*4: Demonstration that outcome of interest was not present at start of study.*

*Outcome assessment:*

*1: Assessment of outcome.*

*2: Follow-up long enough for outcomes to occur.*

*3: Adequacy of follow up of cohorts.*

*Comparability: study controls for the most important factor or any additional factor.*

## Results

### Literature Search Results

**FIGURE 1:**
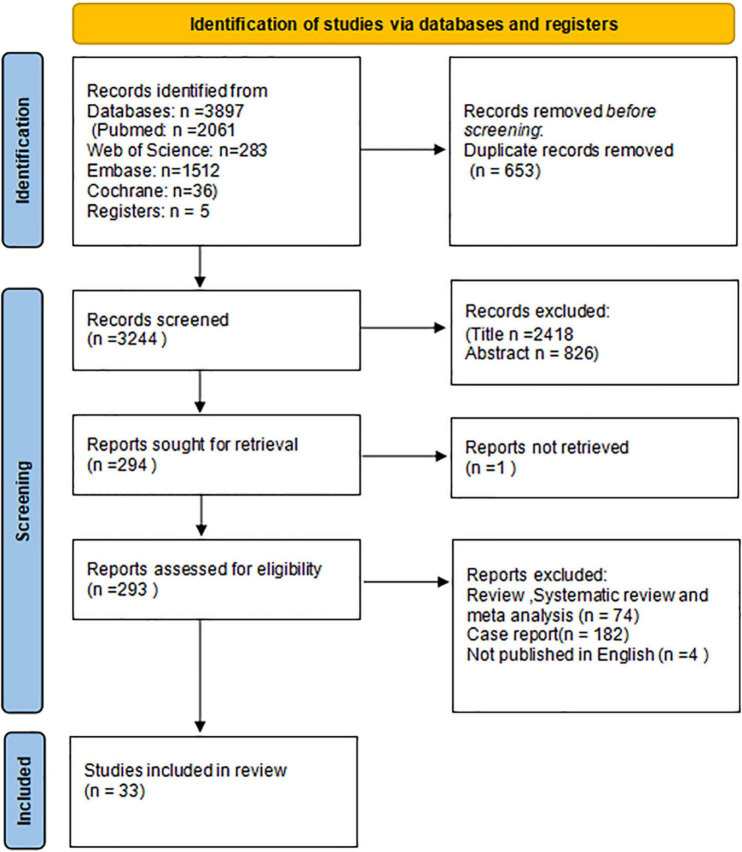
Preferred reporting items for systematic reviews and meta-analyses (PRISMA) flow diagram.

We initially searched for 3,897 results, the process was revealed in the PRISMA flow diagram (Figure 1). The search retrieved 2,061 results from Pubmed, 283 results from the Web of Science, 1,512 results from Embase, 36 results from Cochrane, and five results from Registers. All articles were imported into Endnote 20 (Clarivate Analytics) for screening after removing 653 duplicate records. After the first round by reviewing the title and abstracts, 3,244 studies were removed. Of the 294 reports sought for retrieval, one was not retrieved. In the second round, 293 reports were assessed for eligibility, 74 results were reviews, systematic reviews, and meta-analysis, 182 results were case reports, and four results were not published in English. Finally, 33 articles were included in the review.

### Study Characteristics

A total of 33 articles were included (Table 2), the included studies were retrospective studies and prospective studies, which involve two multicenter studies. In the included literature, not all patients undergoing ERCP were treated with endoscopy simultaneously. Some of them are just diagnostic, and others are followed by other treatments such as surgery after diagnosis. In contrast, some of the patients underwent multiple endoscopic therapeutic procedures during one treatment. Therefore, a total of 13,053 therapeutic ER were performed in 14,162 children. The median patient number in each study is 396, and it ranges from 12 to 11,060. Most studies were conducted on minimally invasive therapy for the HPB system, whose indications include all pediatric hepatic, pancreatic, and biliary diseases. Other studies were only conducted on a single condition, such as pancreatic diseases, symptomatic PBM, and complications after liver, intestine, or composite abdominal organ transplantation. The studies cover Occident (the United States, France, Belgium, etc.) and Orient (China, Japan, South Korea, etc.) countries. A total of 20 studies have been conducted in the last decade, and the other 13 studies were performed before that. Seven articles did not report the total number of ERCP procedures, eight articles did not report the procedural success rates, and three articles did not report the adverse events rates. A total of 24 papers present the classification of specific therapeutic methods, and only 10 of them clarify the percentage of each treatment in all therapeutic procedures (Table 3). Due to the diversity of disease classification and complexity of specific treatment, sex and age stratification were not included in the study.

**TABLE 2:** Characteristics of included studies.

Citation	Country	Study design	Study population *n*	Disease	Number of therapeutic ERCP-procedures % (*n*) (% of ERCP)	Procedural success rates % (*n*)	Adverse events rates % (*n*)
Barakat et al. (23)	United States	Retrospective study	11060	HPB diseases	9456/11060 (85.49%)	N/A	N/A
Mercier et al. (11)	France and Belgium	Retrospective multicentre cohort study	271	HPB diseases	423/470 (90%)	60% (193/320)	19% (65/340)
Lin et al. (24)	United States	Retrospective study	27	PD	58	65% (13/20)	21% (12/58).
Asenov et al. (25)	Turkey	Retrospective study	24	HPB diseases	17/35 (49%)	71% (17/24)	4% (1/25)
Harputluoglu et al. (26)	Turkey	Retrospective study	49	Biliary complications after duct-to-duct biliary anastomosis in LT	49	63.3% (31/49)	N/A
Wen et al. (27)	China	Retrospective study	38	PD presenting with AP/CP	74	93.2% (69/74)	14.9% (11/74)
Zeng et al. (28)	China	Retrospective multicenter study	75	Symptomatic PBM	112	82.4% (56/68)	75%
Kohoutova et al. (29)	Italy	Retrospective study	38	CP	119/158 (75.3%)	74%	3%
Czubkowski et al. (13)	Poland	Retrospective study	30	Biliary strictures after pediatric LT	95	73% (22/30)	17.9% (17/95)
Rosen et al. (30)	United States	Retrospective cohort study	184	HPB diseases	168/215 (78%)	N/A	10.4% (22/212)
Giefer et al. (31)	United States	Retrospective study	276	HPB diseases	345/425 (81.3%)	N/A	7.7% (26/338)
Dechêne et al. (32)	United States	Retrospective study	17	Biliary complications after LT	13/61 (21.3%)	N/A	23.5%
Kargl et al. (33)	Austria	Prospective study	12	Hereditary pancreatitis	25/30 (83.3%)	83.3% (10/12)	16.7% (2/12)
Agarwal et al. (34)	India	Retrospective study	172	Pancreatic disorders	205/221 (92.8%)	64.9%	4.7%
Oracz et al. (35)	Poland	Retrospective study	208	CP	223/481 (46.4%)	98.7% (475/481)	1.9% (9/481)
Steen et al. (36)	Netherlands	Retrospective study	13	Biliary complications after partial liver resection	10	60%	10.8%
Tsuchiya et al. (16)	Japan	Prospective study	55	CC	13/55 (23.6%)	84.6%	N/A
Limketkai et al. (37)	United States	Retrospective cross-sectional study	154	HPB diseases	247/289 (85.5%)	90.7%	5.9%
Enestvedt et al. (38)	United States	Retrospective study	296	HPB diseases	275/429 (64.1%)	95.2%	17.5%
Otto et al. (39)	United States	Retrospective study	25	HPB disease following abdominal organ transplant	42/48 (87.5%)	N/A	2.08%
Berquist (40)	United States	Retrospective study	25	HPB complications after liver, intestine, or composite visceral transplantation	37/48 (77%)	N/A	2.9%
Otto et al. (41)	United States	Retrospective study	167	HPB diseases	159/231 (68.8%)	N/A	4.76% (11/231)
Jang et al. (42)	South Korea	Retrospective study	122	HPB diseases	190/245 (77.6%)	98.4% (241/245)	18.3% (45/245)
Li et al. (18)	China	Retrospective study	51	CP	110	71.4% (30/42)	17.3% (19/110)
Vegting et al. (43)	Netherlands	Retrospective study	61	HPB diseases	60/99 (60.6%)	71% (70/99)	4% (4/99)
Issa et al. (17)	Saudi Arabia	Retrospective study	125	HPB diseases	63/122 (51.9%)	96.8%	3.2%
Rocca et al. (1)	Italy	Retrospective study	38	HPB diseases	33/48 (68.75%)	97.9%	6% (3/48)
Cheng et al. (44)	United States	Retrospective study	245	HPB diseases	235/329 (71.4%)	97.9%	9.7% (32/329)
Varadarajulu et al. (45)	England	Retrospective case-controlled study	116	HPB diseases	110/163 (67.4%)	97.5% (161/163)	3.4% (3/163)
Pfau et al. (46)	United States	Retrospective study	43	HPB diseases	24/53 (45%)	94.3%	12.5% (3/24)
Poddar et al. (47)	India	Retrospective study	72	HPB diseases	22/84 (26.2%)	N/A	8% (6/75)
Hsu et al. (48)	United States	Retrospective study	22	Pancreatitis	23/34 (67.6%)	73.3% (11/15)	6% (2/34)
Guelrud et al. (49)	venezuela	Retrospective study	51	Idiopathic recurrent pancreatitis	18/37 (49%)	83% (15/18)	16.6% (3/18)

*HPB, hepato-pancreato-biliary; PD, pancreas divisum; LT, liver transplantation; AP, acute pancreatitis; CP, chronic pancreatitis; PBM, pancreaticobiliary maljunction; CC, choledochal cysts.*

**TABLE 3:** Random-effects meta-regression to investigate the reason for heterogeneity of the percentage of therapeutic interventions in all endoscopic retrograde cholangiopancreatography (ERCP) procedures.

_meta_es	Coef.	Std. err.	*z*	*P* > | *z*|	[95% Confidence interval]
Country subtype	0.0597887	0.0386503	1.55	0.122	−0.01596450.1355418
Disease subtype	0.0097363	0.0140803	0.69	0.489	−0.01786050.0373331
Year subtype	−0.0744469	0.0267981	−2.78	0.005	−0.1269702–0.0219237
Study design subtype	0.0334285	0.0718178	0.47	0.642	−0.10733170.1741887
_cons	0.7616619	0.0937192	8.13	0.000	0.57797580.9453481

*Test of residual homogeneity: Q_res = chi2(5) = 5.47 Prob > Q_res = 0.3618.*

*Number of obs = 10.*

*Method: REML.*

*Residual heterogeneity:*

*tau2 = 0.000253.*

*I2 (%) = 16.62.*

*H2 = 1.20.*

*R-squared (%) = 78.08.*

*Wald chi2(4) = 10.07.*

*Prob > chi2 = 0.0393.*

*Country subtype:*

*1: Occident countries (the United States, France, Belgium, etc.).*

*2: Orient countries (China, Japan, South Korea, etc.).*

*Disease subtype:*

*1: HPB system diseases, which include all pediatric hepatic, pancreatic, and biliary diseases.*

*2: Pancreatic diseases.*

*3: Hepatobiliary diseases.*

*4: Complications after liver, intestine, or composite abdominal organ transplantation.*

*Year subtype:*

*1: Publications in the last 10 years (2012–2022).*

*2: Publications in the last 10–20 years (2002–2012).*

*3: Articles published 20 years ago.*

*Study subtype:*

*1: Retrospective study.*

*2: Prospective study.*

### The Percentage of Therapeutic Interventions in All Endoscopic Retrograde Cholangiopancreatography Procedures

**FIGURE 2:**
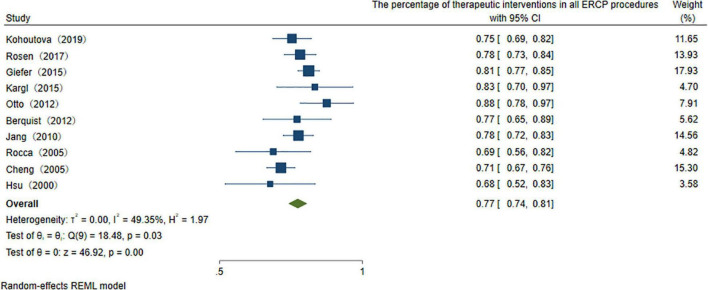
The percentage of therapeutic interventions in all ERCP procedures.

**FIGURE 3:**
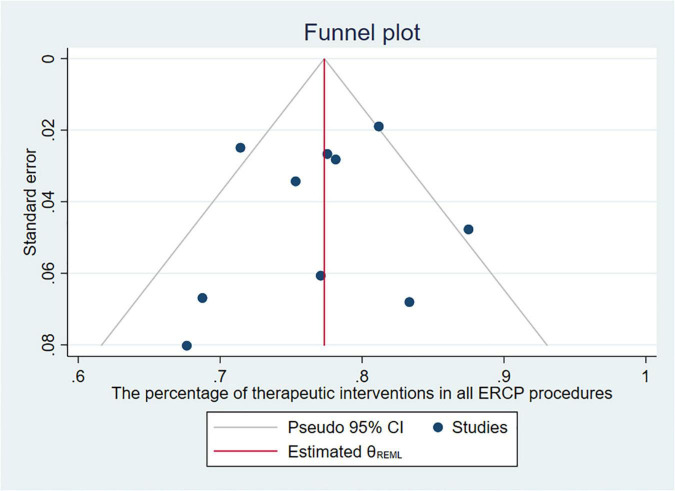
Funnel plot of the percentage of therapeutic interventions in all ERCP procedures.

**FIGURE 4:**
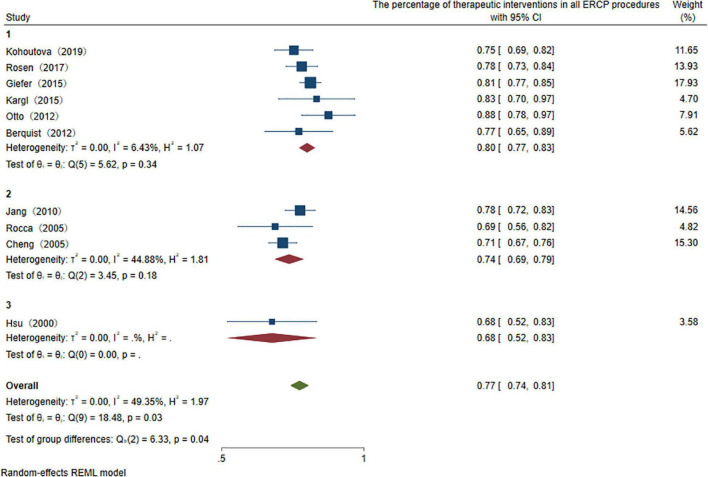
Subtype analysis of publication year of the percentage of therapeutic interventions in all ERCP procedures.

To explore the use ratio of ERCP as a minimal interventional therapy, 26 studies were initially included. After sensitivity analysis, we excluded articles with high heterogeneity, and 10 articles were included finally in the result. The usage rate of therapeutic ERCP is 77% (95% CI 74–81%) (Figure 2). A good symmetry can be roughly seen from the funnel plot, suggesting that there is no significant publication bias. However, some points fall outside the funnel plot, indicating that heterogeneity still exists (Figure 3). Regression-based Egger test for small-study effects shows that *p* = 0.6654, representing no significant publication bias and no small-study effects. This analysis is a confirmation of the stability of our result. Although heterogeneity has been minimized to 49.35%, which is a low heterogeneity, we conduct a meta-regression to investigate the reason for the difference, and the grouping method is shown in Table 3. The results show that the time of publication (year subtype *p* = 0.005) is the cause of heterogeneity. We divided the studies into three subgroups according to publication time: articles published in the last 10 years (2012–2022), articles published in the last 10–20 years (2002–2012), and articles published 20 years ago. We then performed a subgroup analysis on the year of publication and found that heterogeneity was indeed reduced in all groups. The usage rate of therapeutic ERCP is 80% (95% CI 77–83%) in the last decade (2012–2022), which is higher than the other two groups (Figure 4).

### Success Rates and Adverse Event Rates of Therapeutic Procedure

**FIGURE 5:**
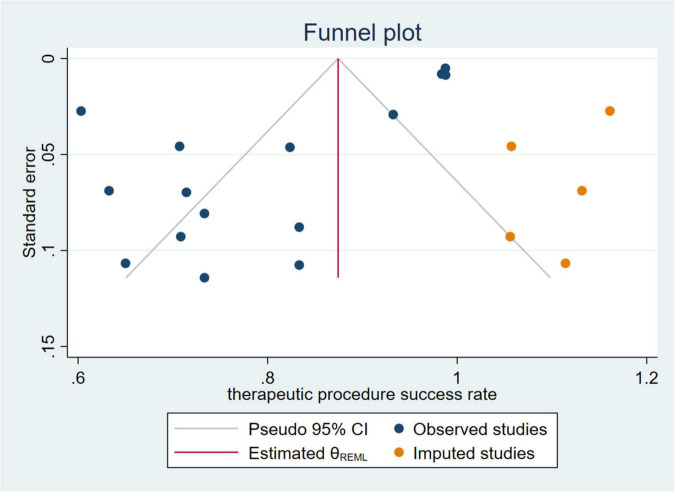
Non-parametric trim-and-fill analysis of publication bias of therapeutic procedure success rate.

**FIGURE 6:**
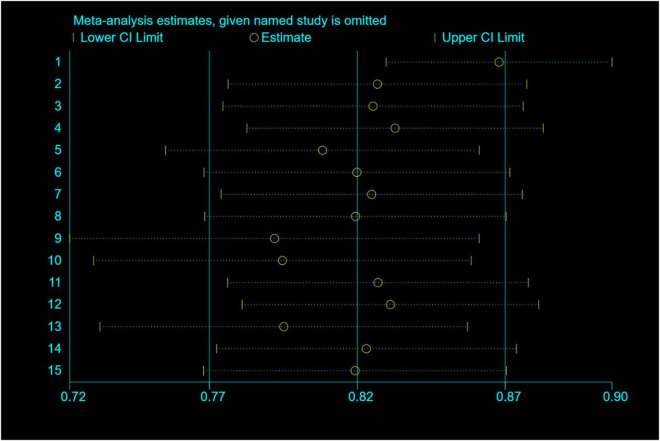
Sensitivity analysis of therapeutic procedure success rate.

**FIGURE 7:**
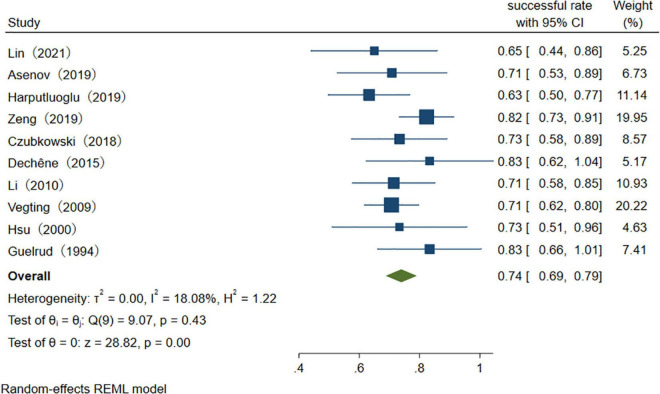
The success rate of therapeutic ERCP procedure.

Among the studies which reported success rates of the therapeutic procedure, the heterogeneity among the originally included studies is significant. To investigate the source of heterogeneity, we performed several studies. We divided the countries into Occident and Orient countries. We divided diseases into overall HPB diseases, pancreatic diseases, biliary diseases, and HPB complications after organ transplantation. The year of publication was divided into the last decade, two decades, and two decades ago. Univariate random-effects meta-regression analysis shows that country (*p* = 0.821), disease (*p* = 0.453), and publication year (*p* = 0.596) are not the source of heterogeneity, multiple meta-regression analyses show that *R*^2^ (%) < 0.001, which indicate that the model constructed by these three variables is invalid. The subgroup analysis showed the same result because the heterogeneity was not well reduced according to the outcome of these three classifications. Non-parametric trim-and-fill analysis of publication bias indicates that after input five studies, the therapeutic procedural success rate is 87.4% (95% CI 79.4–95.4%) (Figure 5). Sensitivity analysis results show that the study has a significant effect on the heterogeneity. Based on the above analysis, five studies were deemed as outlier studies (Figure 6). After the exclusion of the relevant studies, the overall pooled estimates that the therapeutic procedural success rate is 74% (95% CI 69–79%), *I*^2^ = 18.08% (Figure 7).

**FIGURE 8:**
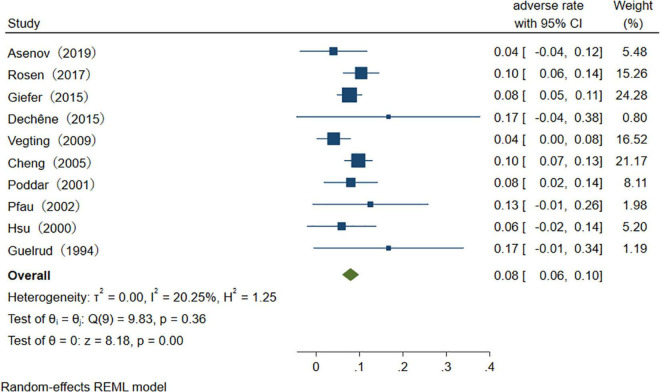
The success rate of therapeutic ERCP procedure.

Of the studies which represent the adverse rates, regression-based Egger test for small-study effects shows that *p* = 0.001, which represents the existence of publication bias. Non-parametric trim-and-fill analysis of publication bias indicates that after input seven studies, adverse rates of the therapeutic procedure are 5.6% (95% CI 2.1–9.1%). After sensitivity analysis, 10 outlier studies were excluded. The overall pooled therapeutic adverse event rate is 8% (95% CI 6–10%), *I*^2^ = 20.25% (Figure 8).

### Distribution of Different Therapeutic Procedures

**FIGURE 9:**
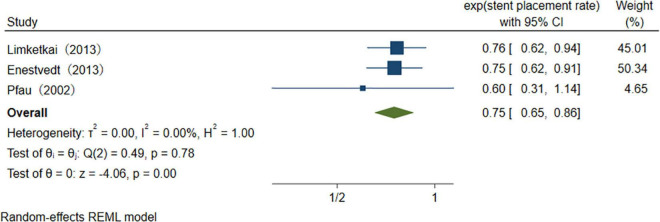
The usage rate of stent placement.

**FIGURE 10:**
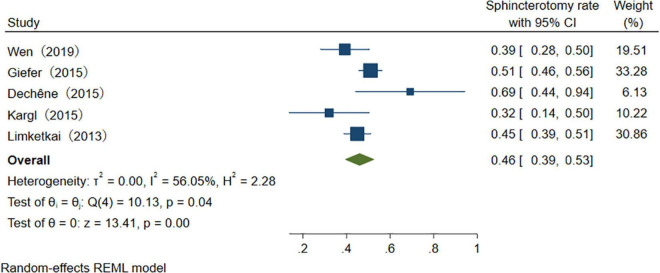
The usage rate of sphincterotomy.

**FIGURE 11:**
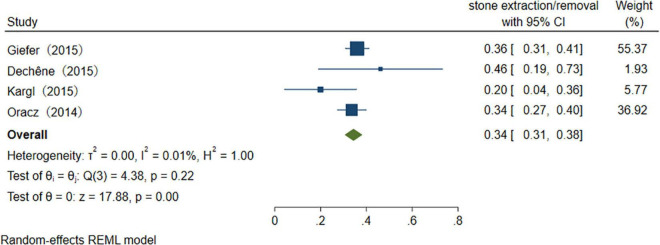
The usage rate of stone extraction/removal.

**FIGURE 12:**
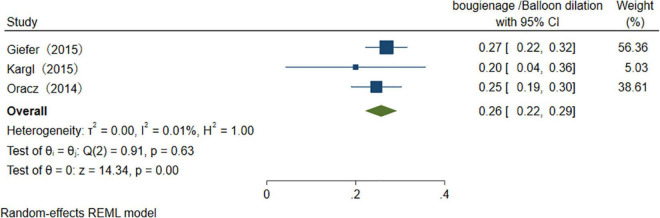
The usage rate of bougienage/Balloon dilation.

With the advancement of endoscopic manipulation techniques, more different treatments are performed in the therapeutic process (Table 4). We analyzed several main treatment methods. The results of three studies reveal that the pooled overall usage rate of stent placement is 75% (95% CI 65–86%), *I*^2^ = 0.00% (Figure 9). Similarly, when pooling studies after excluding papers with high heterogeneity, the usage rate of sphincterotomy (ST), stone extraction/removal, bougienage/balloon dilation was found to be, respectively, 46% (95% CI 39–53%), *I*^2^ = 56.5%, 34% (95% CI 31–38%), *I*^2^ = 0.01%, and 26% (95% CI 22–29%), *I*^2^ = 0.01% (Figures 10–12), as demonstrated in the forest plot.

**TABLE 4:** Distributions of various therapeutic procedures.

	Sphincterotomy	Stone extraction/Removal	Stent placement	Bougienage/Balloon dilation	Bile/Pancreatic duct drainage
Wen et al. (27)	39.2% (29/74)	4.0% (3/74)	10.8% (8/74)	29.7% (22/74)	N/A
Kargl et al. (33)	32% (8/25)	20% (5/25)	32% (8/25)	20% (5/25)	N/A
Giefer and Kozarek (31)	51% (176/345)	35.9% (124/345)	58.3% (201/345)	27% (93/345)	N/A
Dechêne et al. (32)	69.2% (9/13)	46.2% (6/13)	76.9% (10/13)	92.3% (12/13)	N/A
Oracz et al. (35)	31.4% (70/223)	33.6% (75/223)	100% (223)	24.7% (55/223)	N/A
Limketkai et al. (37)	44.9% (111/247)	28.3% (70/247)	43.3% (107/247)	7.7% (19/247)	N/A
Enestvedt et al. (38)	68% (187/275)	40.7% (112/275)	42.9% (118/275)	13.8% (38/275)	N/A
Cheng et al. (44)	76.2% (179/235)	18.3% (43/235)	57% (134/235)	1.7% (4/235)	2.1% (5/235)
Pfau et al. (46)	95.8% (23/24)	58.3% (14/24)	37.5% (9/24)	N/A	N/A
Poddar et al. (47)	4.5% (1/22)	N/A	22.7% (5/22)	4.5% (1/22)	72.7% (16/22)

*n, number; N/A, not available.*

## Discussion

Advances in treatment technology and appropriately sized special endoscopes contribute to the expanded utilization in pediatric HPB diseases. More and more cohort studies and case reports on children have been described in this field. However, data on ERCP therapy is still scarce due to the technical difficulty of performing the intervention in pediatric cohorts and there is no comprehensive study due to the historical limitation of the research. Therefore, the efficacy and safety are still contentious. To guide the further development of clinical work, the therapeutic role of ERCP in children should be demonstrated. ERCP is generally accepted as a therapeutic technique, the proportion of therapeutic ERCP in all procedures is 77% demonstrated in our study. Subtype analysis shows that the proportion of use is increasing every year. In the last decade, it is roughly 80% of total usage, 74% in the last 10–20 years, and only 68% 20 years ago. It reflects that this operation modality has a unique clinical value and is being used by more clinical centers in treating pancreaticobiliary disorders in children.

There is a significant difference between children and adults in the type of anesthesia and the type of duodenoscope (6). ERCP in small children was often performed under general anesthesia with endotracheal intubation, to guarantee satisfaction of this population (7). With technological improvements in non-invasive imaging studies, it is gradually replaced by magnetic resonance cholangiopancreatography (MRCP). Though this cross-sectional imaging modality has no ionizing radiation and does not need intravenous contrast, it still has limitations. For example, it has a complementary lower resolution to discern detailed information such as minor ductal anatomy and abnormalities compared with ERCP, especially in pediatric patients (8). Currently, ERCP is mostly chosen as a therapeutic approach allowing the direct visualization of a biliary pancreatic duct structure. Specialized fiber-optic duodenoscopes are generally used in patients younger than 1 year (6). A systematic review and meta-analysis of single-balloon enteroscopy- (SBE-) assisted ERCP on adult biliary interventions reported that pooled procedural success rate was 75.8% (95% CI 71.0–80.3%) (9). Its feasibility in pediatric patients with surgically altered gastrointestinal anatomy has also been reported recently (10). Therapeutic ERCP procedures may be a beneficial and alternative treatment for HPB diseases, whose success rate in our study is 74% (95% CI 69–79%), especially for pediatric patients, because there are considerable risks for them during surgical operations. By analyzing the influencing factors, country, disease, and publication year did not affect the rate of therapeutic success. Using sensitivity analysis, we found that most of the studies that improve heterogeneity have a larger sample size. The study by Mercier et al. (11), which has the greatest heterogeneity, is a recently published large sample multicenter cohort study. In this study, ERCP was therapeutic in 90% of the included cases, and the overall complication rate was 19% (11). Compared with other included studies, it may have represented the rapid development of the therapeutic ERCP technique, the comprehensive results of multiple diseases, and the proficiency of doctors in French and Belgian children’s medical centers.

In the process of discussing the heterogeneity of procedural success rate by disease subtype analysis, we found *I*^2^ of HPB complications after the organ transplantation group is only 23.87%. This suggests that there may be a subgroup of diseases that benefit similarly from therapeutic ERCP. BC remain relatively common in the pediatric population after liver transplantation, with a wide range of 6–40% (12). Despite a progress in surgical procedures, anastomotic biliary strictures (ABS), leaks, and other complications often cause graft loss and high morbidity. The type of liver graft or abdominal organ transplant may cause different complications. Endoscopic biliary stenting (EBS) drainage with fully covered self-expandable metal stents (FC-SEMSs) or plastic stent (PS) has been reported to be potentially efficient for ABS after liver transplantation in adults (12), and approximately 10–20% of patients require surgical revision in the long run (13). When access to strictures in the peripheral biliary tree is not possible with ERCP, the Rendezvous technique (PTBD + ERCP) may be useful to insert the stent through the stricture (14). Patients with clinical symptoms initially receive an abdominal ultrasound to evaluate the sign of the presence of biliary tract strictures, then a cholangiogram by PTC can demonstrate the presence of caliber changes. During PTC dilation, the stent placement can be performed. If this is not successful, dilation and stent placement can be performed by ERCP.

The adverse event rate of ERCP is higher when the procedure includes a therapeutic intervention than when it is used for diagnostic purposes. In our study, the pooled therapeutic adverse event rate is 8%, which is higher than the overall complication rate of 6% (15). ERCP is less invasive than surgical therapy for the reason that the treatment is less traumatic, and the admissions were shorter, which did not impair school attendance or other activities. However, some patients still required surgical interventions after therapeutic ERCP, these malpractice cases after ERCP manifest that this treatment was not indicated. In these cases, patients suffered a secondary operation injury, and were at risk of adverse events, hence direct surgery would become less invasive and more time-saving. Reasons for subsequent surgery after therapeutic ERCP are: technical or endoscopic therapy failure (failure of cannulation via the minor papilla or failure of pancreatic duct stone extractions), unsatisfactory results of therapy (intractable and recurrent pain), significant comorbidities (intestinal perforation, duodenal hematoma, acute liver failure, progressive graft injury, etc.), complications (refractory bile duct stricture, bile duct obstruction, significant stenosis of the pancreatic duct, etc.), and specific primary diseases (choledocholithiasis, acute gallstone pancreatitis, chronic idiopathic pancreatitis, biliary atresia, etc.) (Table 5). Pre-operative ERCP before surgery may benefit patients with HPB, it can serve as a transitional step to definitive surgery by relieving clinical symptoms (16, 17). Therapeutic management of HPB diseases by ERCP before laparoscopy is effective in many conditions. It provides a more precise visualization of the anatomical structure, which can help during surgical excision. However, surgery is preferred as an effective modality in certain cases, such as established cystic dilation, because chronic inflammation of the bile duct due to reciprocal pancreaticobiliary reflux is a suspicion of malignancy. Although endoscopic treatment can be considered for most symptomatic patients and the cancelation rate was the lowest (23.0%) in adult patients (18), most pediatric surgeons would recommend surgery. For cases where primary pathology needs to be identified and cases with an unknown underlying cause, surgery is also necessary.

**TABLE 5:** Cases required surgical interventions after therapeutic endoscopic retrograde cholangiopancreatography (ERCP).

Study	
Mercier et al. (11)	Additional treatments were needed in 12% (49/394) of cases: surgery in 61% of those cases. Cholecystectomy was performed on 64% (56/87) of the patients who needed an ERCP for choledocholithiasis.
Asenov et al. (25)	2 patients (8%) underwent surgical treatment for not achieving the therapeutic effect. Patient 1 was a 13-year-old boy, PD was found to be the cause. ST of the minor papilla was performed. The effect of the procedure was not permanent, and the patient underwent surgery. Patient 2 with a CBD stone of 2-cm diameter. The therapeutic effect was not achieved. The procedure was abandoned due to limited space for maneuvering the duodenoscope and lithotripter in the duodenum.
Harputluoglu et al. (26)	Endoscopic retrograde cholangiopancreatography (ERCP) and transhepatic biliary interventions (PTBI) were not successful in only 1 living donor liver transplantation (LDLT) patient with stricture. This patient underwent surgical treatment for biliary complications.
Wen et al. (27)	A 10-year-old girl who had CP. MRCP findings: Dorsal duct dilation, Pancreatic stone. This patient underwent pancreaticojejunostomy for failing cannulation *via* the minor papilla.
Kohoutova et al. (29)	1 patient underwent subsequent surgery (hepaticojejunoanastomosis) for refractory bile duct stricture.
Czubkowski et al. (13)	5 patients underwent hepaticojejunostomy and 3 patients required retransplantation (ReLTx). 1 intramural duodenal hematoma requiring surgery. 2 of these patients had important risk factors such as ABO incompatible donor, autoimmune hepatitis (AIH) relapse, and HBV coinfection.
Dechêne et al. (32)	A 13-month-old child required surgical revision for symptomatic duodenal hematoma 48 h after ERCP.
Kargl et al. (33)	2 (2/12) patients with significant stenosis of the pancreatic duct in whom cannulation and stenting were technically impossible underwent open surgical drainage procedures.
Agarwal et al. (34)	2 (2/147) underwent a surgical drainage procedure. Patient 1 had a severe refractory main pancreatic duct head (MPD) stricture. Patient 2 had extensive large PD calculi that were not amenable to extracorporeal shock wave lithotripsy (ESWL).
Oracz et al. (35)	10 patients underwent surgery after ERCP because of unsatisfactory results of stenting therapy. Subtotal pancreatectomy was performed in 3 children, and pancreatic tail resection with Roux-en-Y internal drainage was conducted in 7 cases.
Tsuchiya et al. (16)	11 patients who received a stent underwent excision of the extrahepatic bile duct, endoscopic therapy was considered pre-operative management. 1 patient recurred abdominal pain 18 days after endoscopic drainage. The findings during surgery supported the protein plug theory and verified another rare cause of obstruction involving fatty acid calcium stones.
Limketkai et al. (37)	1 patient underwent surgical intervention after 3 ERCP attempts were unsuccessful in extracting a large pancreatic duct stone.
Otto et al. (41)	41 patients underwent laparoscopic (*n* = 35) or open (*n* = 6) cholecystectomy after ERCP. The cholecystectomies included 17 performed for patients who had cholelithiasis without pancreatitis, including 2 cholecystectomies for patients younger than 10 years. The other indications for cholecystectomy after ERCP were acute gallstone pancreatitis (*n* = 10), choledocholithiasis (*n* = 8), chronic idiopathic pancreatitis (*n* = 4), chronic cholecystitis (*n* = 1), and biliary atresia (*n* = 1). 6 patients required exploratory laparotomy: 5 for drainage or debridement of pancreatic pseudocysts and 1 for excision of a choledochal cyst. 6 patients underwent Roux-en-Y hepaticojejunostomy after ERCP for bile duct obstruction: 3 for pancreatic pseudocyst and 1 each for choledochal cyst, cholangitis, and biliary atresia. Distal pancreatectomy was performed in 3 cases: traumatic pancreatitis in 2 cases and acute pancreatitis with pseudocyst in 1 case.
Jang et al. (42)	Intestinal perforation developed in 2 patients. 1 with perforation of the CC wall and bile leakage, underwent an emergency operation. Bile duct dilation improved in 2 such patients, both of whom underwent laparoscopic cholecystectomy.
Li et al. (18)	5 (11.6%) patients received surgical interventions, including pancreaticojejunostomy procedure (*n* = 3), Leger’ s procedure (*n* = 1), and “pancreatic head resection” in another hospital (*n* = 1) because of intractable pain (*n* = 1) and recurrent pain due to failure of pancreatitic duct stone extractions (*n* = 4). All five patients had histological evidence of CP.
Issa et al. (17)	36 patients had ERCP with sphincterotomy and stone extraction and 34 of them subsequently underwent laparoscopic cholecystectomy. This sequential approach is safe and effective for the management of children with cholelithiasis and choledocholithiasis.
Varadarajulu et al. (45)	A 9-year-old child with annular pancreas and chronic pancreatitis, the stent could not be inserted through a dominant stricture in the head of the pancreas, and surgery was recommended.

In our study, the pooled overall usage rate of stent placement is 75%. The usage rate of ST, stone extraction/removal, and bougienage/balloon dilation is, respectively, 46, 34, and 26%. EST or biliary drainage by ERCP is relatively minimal invasive and has a shorter operating duration. Ductal drainage is an effective method to redress elevated intra-organic pressure, which can reduce pain and recurrence. The goal of endoscopic interventions is to reduce pressure in the damaged biliary or pancreatic duct system, early drainage is the main prerequisite for spontaneous closure of the leak. Most patients benefit from ST, EPS, or stent placement through pancreaticobiliary duct decompression. Endoscopic drainage of pancreaticobiliary ducts is now accepted as a viable treatment option in a wide range of cases. Ductal obstruction and ductal disruption (pancreaticobiliary duct leak, ductal stricture, and residual stone diseases) can be treated by ERCP stent placement, especially in anatomically complex patients (19). Our study demonstrates that stent placement is the most common usage, which accounts for 75% of all therapeutic procedures. A recent study shows the temporary use of FC-SEMSs for recurrent benign main pancreatic duct (MPD) strictures in children is feasible and safe (20). In many other cases, such as post-traumatic duct disruption, it can also be managed by temporary stenting. Emergent excision may be dangerous under such particular circumstances, except when precise pancreaticobiliary anatomy is obtained (21). Although there are advantages, prophylactic or therapeutic stent placement may be associated with a higher rate of post-ERCP pancreatitis (PEP), which is demonstrated by a univariate analysis (11). Long-term management requires stent exchange.

It is important to make the proper choice of endoscopic therapy, surgery, and other treatments. More well-designed, large clinical controlled trials are necessary to be performed in this field. There should be evidence-based guidelines to recommend the therapeutic ERCP procedure, which will specifically benefit certain types of pediatric patients. Endoscopists and surgeons who specialized in evaluating pediatric patients usually function as members of a multidisciplinary team. To yield better outcomes and to decrease the number of hospitalizations and surgical interventions, every step of the process, from preparation to subsequent treatment, should be performed in close collaboration with anesthesiologists, pediatricians, pediatric surgeons, and gastroenterologists.

The limitations of our study should be acknowledged: the outcomes of pediatric ERCP performed by surgical vs. medical specialties are different. Small vs. large patient volume healthcare facilities should be compared. Robust volume outcomes should be obtained from a larger population. Pediatric ERCP had different roles in disparate age groups, so age stratification is also a potential influence factor (22). A comprehensive assessment of these data may help expand the awareness of doctors in the usage of minimally invasive techniques to treat HPB diseases and contribute to the improvement of therapeutic methods and enhance the utilization rate in this field.

Our analysis indicates that ERCP is an effective treatment option for pediatric HPB diseases. Given the above analysis, we deem it reasonable to perform therapeutic ERCPs under appropriate conditions to achieve long-term remission. Further study should be conducted to decipher the feasibility of therapeutic ERCP in children.

## Data Availability Statement

The original contributions presented in this study are included in the article/Supplementary Material, further inquiries can be directed to the corresponding author.

## Author Contributions

RS and XX made substantial contributions to the conception or design of the work and the acquisition, analysis, or interpretation of data for the work, and screened all articles relevant to therapeutic ERCP for Pediatric HPB diseases for methodological validity. QZ resolved the discordance during the study and drafted the work or revised it critically for important intellectual content. RS and QZ assessed the quality measures for included studies and discrepancies were adjudicated *via* consensus or referral to XX. JZ gave approval of the final version to be published. All authors evaluated firstly the titles, abstracts, and keywords and after the initial screening full texts of the manuscript were retrieved and evaluated for eligibility, agreed to be accountable for all aspects of the work in ensuring that questions related to the accuracy or integrity of any part of the work are appropriately investigated and resolved, contributed to the article, and approved the submitted version.

## Conflict of Interest

The authors declare that the research was conducted in the absence of any commercial or financial relationships that could be construed as a potential conflict of interest.

## Publisher’s Note

All claims expressed in this article are solely those of the authors and do not necessarily represent those of their affiliated organizations, or those of the publisher, the editors and the reviewers. Any product that may be evaluated in this article, or claim that may be made by its manufacturer, is not guaranteed or endorsed by the publisher.
