# Supplementary material for: Therapeutic Endoscopic Retrograde Cholangiopancreatography for Pediatric Hepato-Pancreato-Biliary Diseases: A Systematic Review and Meta-Analysis
Source: Front Pediatr. 2022 Jun 30;10:915085. doi: 10.3389/fped.2022.915085 (PMC9280719; doi:10.3389/fped.2022.915085)
Supplement: Supplementary file 1 [file Table_1.docx]

Therapeutic ERCP

PubMed

“Pediatrics”[Mesh] OR “Child”[Mesh] OR “Adolescent”[Mesh] OR “Infant”[Mesh] OR paediatri*[Title/Abstract] OR pediatri*[Title/Abstract] OR adolesc*[Title/Abstract] OR pubescen*[Title/Abstract] OR juvenil*[Title/Abstract] OR child*[Title/Abstract] OR boy*[Title/Abstract] OR girl*[Title/Abstract] OR minors*[Title/Abstract] OR school*[Title/Abstract] OR student*[Title/Abstract] OR teen*[Title/Abstract] OR young*[Title/Abstract] OR youth*[Title/Abstract] OR preschool*[Title/Abstract] OR pre-school*[Title/Abstract] OR infan*[Title/Abstract] OR baby*[Title/Abstract] OR minor*[Title/Abstract] OR newborn*[Title/Abstract] OR neonat*[Title/Abstract] OR toddler[Title/Abstract]

“Cholangiopancreatography, Endoscopic Retrograde”[Mesh] OR Retrograde Cholangiopancreatography, Endoscopic[Title/Abstract] OR Cholangiopancreatographies, Endoscopic Retrograde[Title/Abstract] OR Endoscopic Retrograde Cholangiopancreatographies[Title/Abstract] OR Retrograde Cholangiopancreatographies, Endoscopic[Title/Abstract] OR Endoscopic Retrograde Cholangiopancreatography[Title/Abstract] OR ERCP[Title/Abstract]

“Therapeutics”[Mesh] OR Therapeutic[Title/Abstract] OR Therapy[Title/Abstract] OR Therapies[Title/Abstract] OR Treatment[Title/Abstract] OR Treatments[Title/Abstract]

WOS

TS=(“Pediatrics” OR “Child” OR “Adolescent” OR “Infant” OR paediatri* OR pediatri* OR adolesc* OR pubescen* OR juvenil* OR child* OR boy* OR girl* OR minors* OR school* OR student* OR teen* OR young* OR youth* OR preschool* OR pre-school* OR infan* OR baby* OR minor* OR newborn* OR neonat* OR toddler)

TS=(“Appendicitis” OR Appendicitis* OR Ruptured Appendicitis OR Perforated Appendicitis

)

TS=(“Cholangiopancreatography, Endoscopic Retrograde” OR Retrograde Cholangiopancreatography, Endoscopic OR Cholangiopancreatographies, Endoscopic Retrograde OR Endoscopic Retrograde Cholangiopancreatographies OR Retrograde Cholangiopancreatographies, Endoscopic OR Endoscopic Retrograde Cholangiopancreatography OR ERCP)

TS=(“Therapeutics” OR Therapeutic OR Therapy OR Therapies OR Treatment OR Treatments)

Embase

'pediatrics'/exp OR 'juvenile'/exp OR 'adolescent'/exp OR 'child'/exp OR 'boy'/exp OR 'brain damaged child'/exp OR 'girl'/exp OR 'handicapped child'/exp OR 'hospitalized child'/exp OR 'infant'/exp OR 'baby'/exp OR 'high risk infant'/exp OR 'hospitalized infant'/exp OR 'newborn'/exp OR 'preschool child'/exp OR 'school child'/exp OR 'toddler'/exp

paediatri*:ab,ti OR pediatri*:ab,ti OR adolesc*:ab,ti OR pubescen*:ab,ti OR juvenil*:ab,ti OR child*:ab,ti OR boy*:ab,ti OR girl*:ab,ti OR minors*:ab,ti OR school*:ab,ti OR student*:ab,ti OR teen*:ab,ti OR young*:ab,ti OR youth*:ab,ti OR preschool*:ab,ti OR 'pre school*':ab,ti OR infan*:ab,ti OR baby*:ab,ti OR minor*:ab,ti OR newborn*:ab,ti OR neonat*:ab,ti OR toddler:ab,ti

'endoscopic retrograde cholangiopancreatography'/exp

'cholangiopancreatography, endoscopic retrograde':ab,ti OR e.r.c.p.:ab,ti OR 'endoscopic retrograde pancreaticocholangiography':ab,ti OR ercp:ab,ti OR 'pancreatocholangiography, endoscopic retrograde':ab,ti OR 'retrograde endoscopic cholangiopancreatography':ab,ti

'Therapeutics'/exp

Therapeutic:ab,ti OR Therapy:ab,ti OR Therapies:ab,ti OR Treatment:ab,ti OR Treatments:ab,ti

Cochrane

(paediatri* OR pediatri* OR adolesc* OR pubescen* OR juvenil* OR child* OR boy* OR girl* OR minors* OR school* OR student* OR teen* OR young* OR youth* OR preschool* OR pre-school* OR infan* OR baby* OR minor* OR newborn* OR neonat* OR toddler):ti,ab,kw

MeSH descriptor: [Pediatrics] explode all trees

MeSH descriptor: [Child] explode all trees

MeSH descriptor: [Adolescent] explode all trees

MeSH descriptor: [Infant] explode all trees

(Retrograde Cholangiopancreatography, Endoscopic OR Cholangiopancreatographies, Endoscopic Retrograde OR Endoscopic Retrograde Cholangiopancreatographies OR Retrograde Cholangiopancreatographies, Endoscopic OR Endoscopic Retrograde Cholangiopancreatography OR ERCP):ti,ab,kw

MeSH descriptor: [Cholangiopancreatography, Endoscopic Retrograde] explode all trees

(drain* OR prophylactic drain* OR paracenteses* OR puncture and aspiration OR aspiration and puncture OR centesis* OR centeses* OR puncture and drainage OR drainage and puncture OR culdocentesis* OR culdocenteses* OR suction* OR aspiration* OR mechanical aspiration* ):ti,ab,kw

MeSH descriptor: [Therapeutics] explode all trees

Trial Registers

ClinicalTrials.gov

Therapeutics | Endoscopic Retrograde Cholangiopancreatography OR ERCP | Child

WHO ICTRP

Endoscopic Retrograde Cholangiopancreatography AND Therapeutics
